# Identification and characterization of a novel thermostable pyrethroid-hydrolyzing enzyme isolated through metagenomic approach

**DOI:** 10.1186/1475-2859-11-33

**Published:** 2012-03-13

**Authors:** Xinjiong Fan, Xiaolong Liu, Rui Huang, Yuhuan Liu

**Affiliations:** 1 School of life sciences, Sun Yat-sen University, Guangzhou 510275, P. R. China; 2 Guangzhou Liby Enterprise CO., LTD, Guangzhou 510170, P. R. China

**Keywords:** Metagenomic library, Esterase, Pyrethroid, Thermostable, Turban basin

## Abstract

**Background:**

Pyrethroid pesticides are broad-spectrum pest control agents in agricultural production. Both agricultural and residential usage is continuing to grow, leading to the development of insecticide resistance in the pest and toxic effects on a number of nontarget organisms. Thus, it is necessary to hunt suitable enzymes including hydrolases for degrading pesticide residues, which is an efficient "green" solution to biodegrade polluting chemicals. Although many pyrethroid esterases have consistently been purified and characterized from various resources including metagenomes and organisms, the thermostable pyrethroid esterases have not been reported up to the present.

**Results:**

In this study, we identified a novel pyrethroid-hydrolyzing enzyme Sys410 belonging to familyV esterases/lipases with activity-based functional screening from Turban Basin metagenomic library. Sys410 contained 280 amino acids with a predicted molecular mass (Mr) of 30.8 kDa and was overexpressed in *Escherichia coli *BL21 (DE3) in soluble form. The optimum pH and temperature of the recombinant Sys410 were 6.5 and 55°C, respectively. The enzyme was stable in the pH range of 4.5-8.5 and at temperatures below 50°C. The activity of Sys410 decreased a little when stored at 4°C for 10 weeks, and the residual activity reached 94.1%. Even after incubation at 25°C for 10 weeks, it kept 68.3% of its activity. The recombinant Sys410 could hydrolyze a wide range of ρ-nitrophenyl esters, but its best substrate is ρ-nitrophenyl acetate with the highest activity (772.9 U/mg). The enzyme efficiently degraded cyhalothrin, cypermethrin, sumicidin, and deltamethrin under assay conditions of 37°C for 15 min, with exceeding 95% hydrolysis rate.

**Conclusion:**

This is the first report to construct metagenomic libraries from Turban Basin to obtain the thermostable pyrethroid-hydrolyzing enzyme. The recombinant Sys410 with broad substrate specificities and high activity was the most thermostable one of the pyrethroid-hydrolyzing esterases studied before, which made it an ideal candidate for the detoxification of pyrethroids.

## Background

Pyrethroid pesticides are synthetic analogues of pyrethrins, which are natural chemicals derived from Chrysanthemum flowers [1]. They are also used as broad-spectrum pest control agents in agricultural production, thanks to their high toxicities to insects and low toxicities to mammals [2]. Currently, organophosphorous pesticides are increasingly being replaced by pyrethroid pesticides, and the impact of the pyrethroid pesticides residual on the environment is likely to draw more attention [3,4]. Both agricultural and residential usage is continuing to grow [5], leading to the development of insecticide resistance in the pest and toxic effects on a number of nontarget organisms, such as man, fish and bees [6-8].

Current disposal methods for pesticides residual are both abiotic and biotic pathways, including photooxidation, chemical oxidation and biodegradation. Moreover, enzymes can offer an efficient "green" solution to biodegrade polluting chemicals, thereby playing pivotal roles in the field of bioremediation [9]. Thus, it is necessary to hunt suitable enzymes including hydrolases for degrading pesticide residues [10-12].

Esterases, a generic term for a hydrolase that catalyzes the cleavage and formation of ester bonds, play a major role in the degradation of natural compounds and industrial pollutants, and thus dominate the industrial market for the food, medicine, biodiesel, and agricultural industries with attractive features [13,14]. Some pyrethroid esterases have consistently been purified and characterized from various resources including metagenomes and organisms [12,15-18]. To date, the thermostable pyrethroid esterases have not been reported.

Thermotolerant or thermophilic microorganisms are a valuable source of thermostable enzymes often associated with stability in solvents and detergents, giving these enzymes considerable potential applications in many industries [19,20]. An approach to obtain novel, efficient and thermostable enzymes from natural resources is gaining momentum. The use of metagenomics, a culture-independent technique [21-23], does not require the cultivation of diverse microorganisms from environmental samples, which is often difficult or impossible. This maximizes our chances to obtain ideal biocatalysts, such as lipases and esterases [24-29]. The Turban Basin, the hottest area in China, has attracted the attention of scholars and scientists with its unique position. The average temperature in summer is beyond 38°C and, interestingly, land surface temperature is more than 82°C for a certain period. Therefore, most of the enzymes released by natural microorganisms in Turban Basin possess thermostable characteristics.

In order to obtain novel thermostable pyrethroid-hydrolyzing enzyme that share low or moderate homology with other known counterparts, we constructed metagenomic library from Turban Basin soil by functional expression screening of plasmid clones with esterase activity. Three clones with esterase activity were detected, but only one novel thermostable pyrethroid-hydrolyzing enzyme was selected for further characterization including thermal stability, substrate specificity and degradation efficiency. To our knowledge, this is the first report so far on information about thermostable pyrethroid-hydrolyzing enzyme gene from the unculturable bacterial genome. Further study is helpful to obtain excellent detoxifying enzyme for bioremediation.

## Results

### Screening for pyrethroid-hydrolyzing esterases from a metagenomic library

The total DNA for metagenomic library was extracted from Turban Basin soil. Approximately 21,000 cells of *Escherichia coli *(*E. coli*) TOP10 containing the pUC118-based metagenomic DNA library were prepared. The average insert size was 4.8 kb, and sizes ranged from 2.5 to 10 kb. The metagenomic library represented about 100 Mb of soil microbial community DNA. Out of approximately 21,000 colonies, 3 clones were identified by their bright blue color. There was only one positive blue colony *E. coli *TOP10 (pUC3) with the ability to hydrolyze pyrethroids confirmed by gas chromatography analysis. Therefore, pUC3 was selected to further research.

### Genetic characterization

The fragment size in plasmid pUC3 was 3.936 kb. The sequence analysis of the insert DNA showed the presence of one open reading frame (ORF) 843 bp, encoding a polypeptide of 280 amino acids with a predicted molecular mass (Mr) of 30.8 kDa. The putative esterase gene was designated *sys410*.

**Figure 1:**
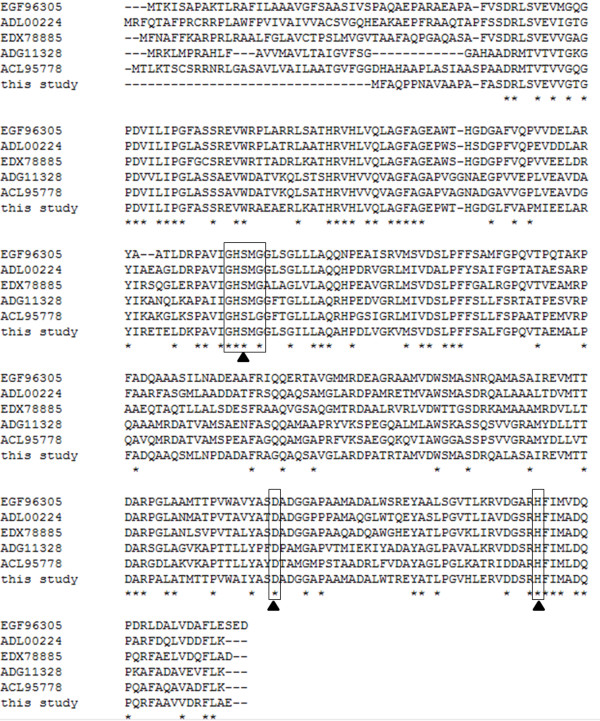
**Multiple amino acid sequence alignment of Sys410**. Multiple alignment of the partial Amino acid sequences containing the conserved motifs of G × S × G and putative catalytic triad resides of alpha/beta hydrolase family proteins. Except for Sys410 (this study), other protein sequences were retrieved from GenBank http://www.ncbi.nlm.nih.gov. The accession numbers of the aligned sequences are for the following organisms: EGF96305, alpha/beta hydrolase fold family protein from *Brevundimonas diminuta *ATCC 11568; ADL00224, alpha/beta hydrolase fold protein from *Brevundimonas subvibrioides *ATCC 15264; EDX78885, hydrolase, alpha/beta fold family from *Brevundimonas sp*. BAL3; ADG11328, alpha/beta hydrolase fold protein from *Caulobacter segnis *ATCC 21756; ACL95778, alpha/beta hydrolase-family protein from *Caulobacter crescentus *NA1000. The alignment was carried out using the Clustal W method. The open boxes indicate amino acid resides belonging to the putative catalytic triad resides, triangles denote the active site. The same amino acid resides are marked by (*).

**Figure 2:**
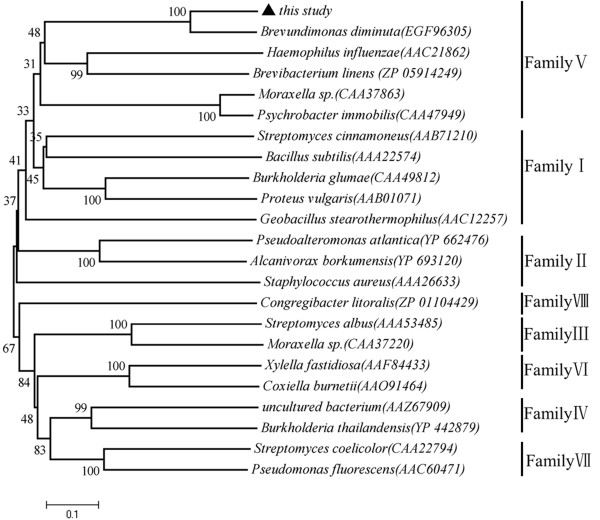
**Phylogenetic analysis of Sys410, its homologues and other esterases**. Phylogenetic relationship of Sys410 and lipase/esterase proteins of eight different families was performed using the program MEGA 5.05. Except for Sys410, the other protein sequences for previously identified families of bacterial lipolytic and esterolytic enzymes were retrieved from GenBank http://www.ncbi.nlm.nih.gov. The numbers at node indicate the bootstrap percentages of 1000 resamples. The units at the bottom of the tree indicate the number of substitution events.

The putative amino acid sequence of Sys410 was used to perform a BLAST program provided by the National Centre for Biotechnology Information and Swissprot databases. This search showed the moderate identity between Sys410 and other esterases/lipases, the highest with alpha/beta hydrolase fold family protein *Brevundimonas diminuta *ATCC 11568 (200/265, 75% identity). Multiple sequence alignment of Sys410 and other lipase/esterase proteins revealed the typical catalytic triad of active site serine (S102) motif G-X-S-X-G (Figure 1), conserved aspartic acid (D226), and histidine (H260) residue motif in the encoded protein [13,30]. Bacterial lipase/esterase proteins have been classified into eight different families on the basis of their amino acid sequences and biochemical properties [31]. A phylogenetic tree was constructed to further verify the evolutionary relationship among Sys410 and other known lipase/esterase proteins [28]. These results suggested that Sys410 belongs to familyV (Figure 2).

### Heterologous expression and purification of recombinant Sys410

**Figure 3:**
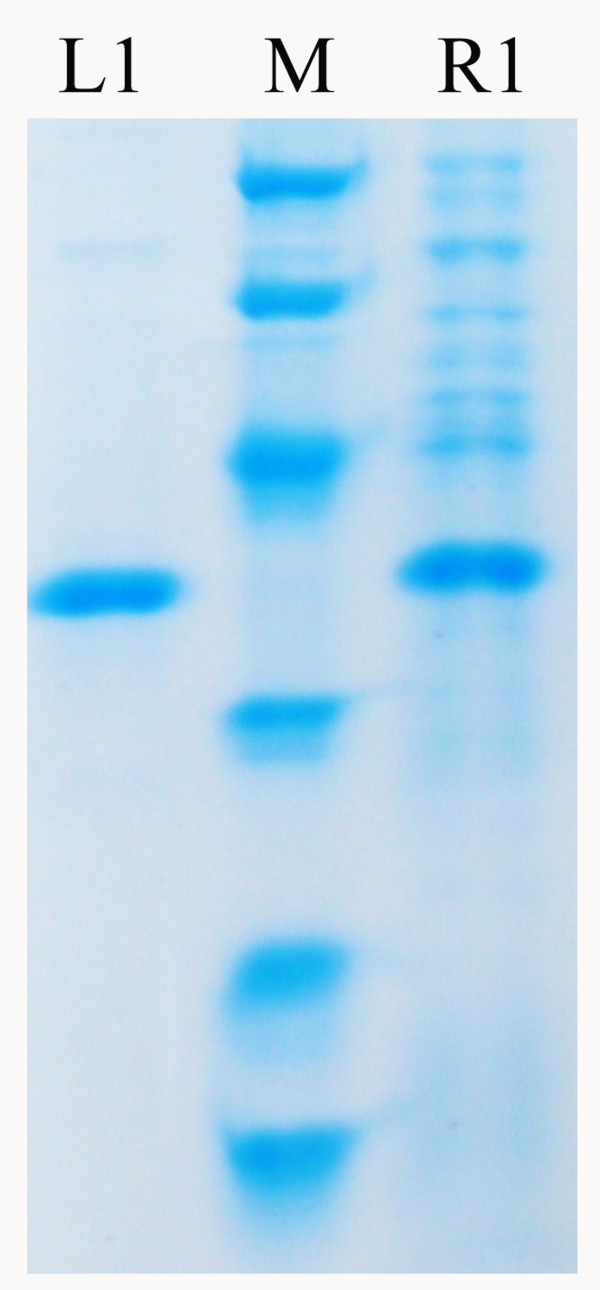
**SDS-PAGE analysis of the purified recombinant Sys410**. Purified target protein (lane L1); supernatant of *E. coli *BL21 (DE3) cell lysates (lane R1); protein markers (lane M) stained with Coomassie blue, with the list from top to bottom 97.2 kDa, 66.4 kDa, 44.3 kDa, 29.0 kDa, 20.1 kDa, 14.3 kDa.

The full-length *sys410 *gene was amplified and cloned into the expression vector pET-28a (+) with a C-terminal 6 × His tag, and expressed in *E. coli *BL21 (DE3) with 0.6 mM IPTG induction at 37°C for 8 h, then purified by Ni-NTA-agarose chromatography. The target recombination protein appeared as a single band on SDS-PAGE with molecular weight 36.7 kDa (Figure 3), consist of the 280 amino acids with a fusion of 54 amino acids corresponding to polyhistidine tag (His-tag), a unique thrombin cleavage site (Thrombin). The highest expression level of Sys410 (OD_600 _= 3.8) was about 0.24 mg/mL and its content in total soluble protein reached up to 50% according to Quantity One software (Bio-Rad laboratories Inc., Hercules, USA) for protein band visualization.

### Effect of pH and temperature on Sys410 activity and stability

**Figure 4:**
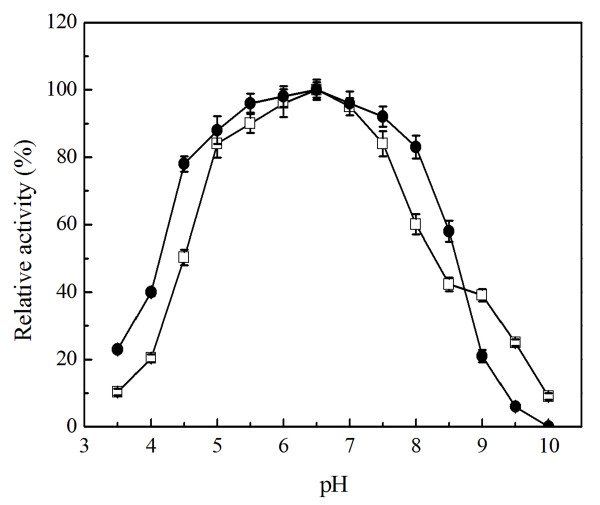
**Effect of pH on activity (■) and stability (●) of recombinant Sys410**. The purified enzyme was preincubated in different buffers for 12 hours at 30°C.

The effect of pH over purified Sys410 activity was determined using ρ-nitrophenyl acetate as a substrate at 40°C. Sys410 displayed high activity at pH values between 5.0 and 7.5, and optimal pH is 6.5 (Figure 4). The pH stability was tested after incubation of purified Sys410 in various buffers at pH 3.5 to 10.0. After 24 h incubation, Sys410 displayed more than 80% residual activity in the pH range 5.0 to 8.0. pH stability was therefore concluded to be greatest at pH 6.5.

**Figure 5:**
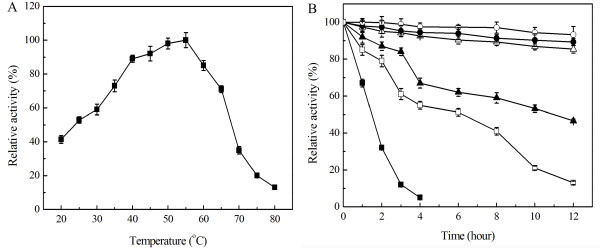
**Effect of temperature on activity (A) and stability (B) of recombinant Sys410**. The purified enzyme was preincubated at 35°C (○), 40°C (●), 45°C (△), 50°C (▲), 55°C (□), 60°C (■) for 12 hours.

The effect of temperature over purified Sys410 activity was made using ρ-nitrophenyl acetate as a substrate at pH 6.5, with temperatures ranging from 20 to 80°C. Esterase activity increased as temperature increased up to 55°C, and decreased beyond that level. At temperatures above 80°C, there was essentially little enzyme activity. The optimal temperature was 55°C (Figure 5). Thermostability was determined by analysis of residual activity at regular intervals after preincubation for durations up to 12 h, at temperatures ranging from 35-60°C. Sys410 was very stable below 45°C, with residual activity exceeding 85% after incubation for 12 h (Figure 5).

**Figure 6:**
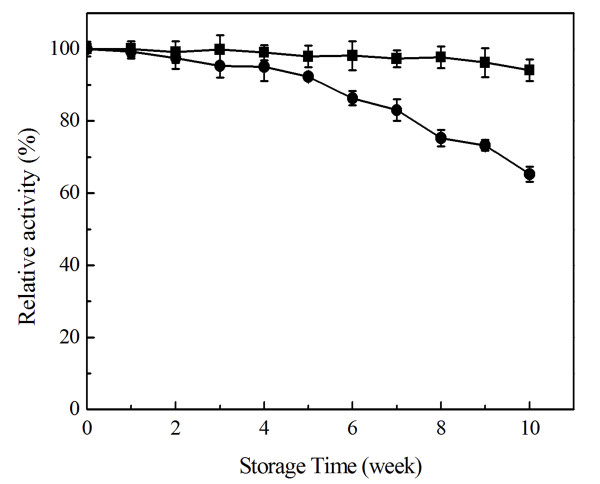
**The storage stability of recombinant Sys410 at 4°C (■) and 25°C (●)**.

The storage stability of the recombinant enzyme was determined at 4°C and 25°C for 10 weeks (Figure 6). Esterase activity decreased a little when stored in 50 mM potassium phosphate buffer (pH 6.5) at 4°C for 10 weeks, and the residual activity reached 94.1%. Sys410 kept 68.3% of its activity after incubation at 25°C for 10 weeks.

### Substrate specificity and activity of Sys410

To determine the substrate specificity of Sys410, we tested its activity on various ρ-nitrophenyl esters with acyl chain lengths of C2, C4, C6, C8, C10, and C12 under assay conditions of pH 6.5 and 55°C. Recombinant Sys410 showed the highest activity with ρ-nitrophenyl acetate (772.9 U/mg) among the ρ-nitrophenyl esters examined, low levels of activity toward ρ-nitrophenyl esters with long acyl groups (Table 1). The *K*m and *k*_cat _values were calculated by fitting the data to Michaelis-Menten equation. Moreover, when acyl chain lengths were C2, its *k*_cat _and *K*m values were 289.1 S^-1 ^and 14.1 μM, respectively. When acyl chain lengths were between 4 and 12, its *k*_cat _values decreased, while *K*m values rose with acyl chain length rising.

**Table 1:** Kinetic characterization of recombinant Pye843

Substrates	*K*m (μM)	*k*_cat _(S^-1^)	Enzyme activity (U/mg)
ρ-nitrophenyl acetate	14.1 ± 3.2	289.1 ± 3.0	772.9 ± 3.4
ρ-nitrophenyl butyrate	31.6 ± 4.0	163.3 ± 6.2	408.3 ± 9.2
ρ-nitrophenyl caprylate	33.2 ± 2.1	149.7 ± 8.3	388.6 ± 5.4
ρ-nitrophenyl caproate	57.6 ± 1.9	88.9 ± 3.5	115.7 ± 4.3
ρ-nitrophenyl decanoate	99.7 ± 9.3	69.1 ± 2.2	56.9 ± 3.2
ρ-nitrophenyl laurate	130.9 ± 7.2	49.1 ± 6.6	34.5 ± 2.1

**Figure 7:**
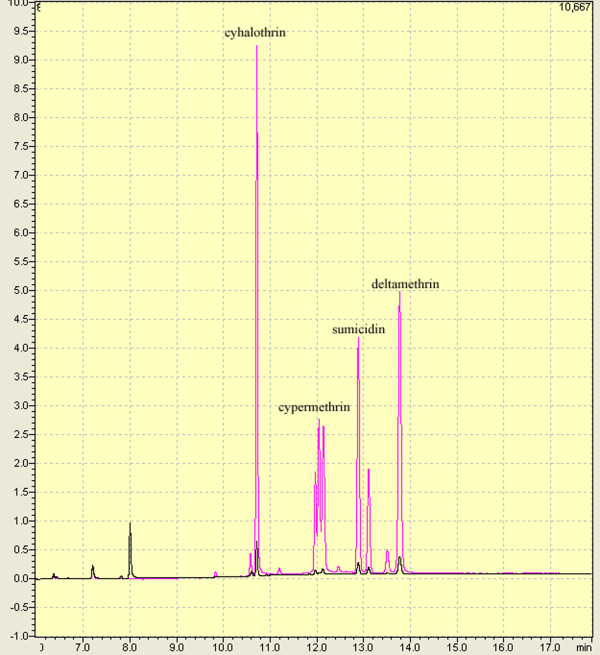
**Gas chromatography analysis of different pyrethroids hydrolyzed by Sys410**. The red line and black line stand for the hydrolysis chromatography by inactive enzyme (control) and active enzyme, respectively.

The degradation efficiency by Sys410 was tested with four different pyrethroids as the substrates by gas chromatography analysis (Figure 7). The hydrolysis rates of cyhalothrin, cypermethrin, sumicidin and deltamethrin were 98.1%, 99.7%, 97.1%, 95.8%, respectively, under assay conditions of pH 6.5 and 37°C for 15 min.

## Discussion

Soil mainly reserves abundant microbial diversity on the earth [32]. According to reports, more than 99% microorganisms can not be cultured [33-37]. Fortunately, the number of potential biocatalysts from uncultured microorganisms is rising with the advent of the metagenomic approach. Therefore, metagenomic libraries should be useful for screening novel thermostable enzymes, including esterases, but no such libraries have been reported yet from thermal environmental soils with land surface temperature 82°C from Turban Basin in China.

In this library of 21,000 clones, 3 blue color transformants were initially selected with the esterase activity in LB agar plates containing 100 μM X-caprylate, and further analysis determined that only one transformant hydrolyzed pyrethroids efficiently. These results showed that not all esterases are capable of degrading pyrethroids. Both the nucleotide and amino acid sequences of the Sys410 enzyme were novel, with the moderate identity between Sys410 and other esterases/lipases.

The novel pyrethroid-hydrolyzing esterase Sys410 had more than 50% activity from 25°C to 65°C, and even displayed 41.4% activity at 20°C. So Sys410 possessed high activity at wide range of temperatures, different from other known pyrethroid-hydrolyzing esterases [12,15,16]. Sys410 also exhibited thermostable property. The enzyme was very stable below 45°C. After 12 h of incubation at 45°C, it remained 85.4% activity. Sys410 had half-lives of approximately 6 h and 10 h at 50°C and 55°C, respectively. Interestingly, even incubating at 60°C for 1 h, it still retained as much as 67.2% of its total activity, and then dropped dramatically, which was different from PytH with 55% residual activity at 60°C for 1 h [15,16]. The residual activity was 94.1% and 68.3% after incubation 4°C and 25°C for 10 weeks, respectively, which indicated that Sys410 displayed high storage stability. These results above showed that Sys410 was the most thermostable one of the pyrethroid-hydrolyzing esterases studied before and more suitable for application.

Generally, there is a negative correlation between *K*m and *k*_cat _values for one enzyme toward different substrates. A low *K*m value for a substrate indicates positive affinity for the enzyme, followed with higher catalytic activity and consequently a higher *k*_cat _value [28]. Sys410 showed a specific preference for ρ-nitrophenyl acetate to other substrates, which had the lowest *K*m value (14.1 μM) and highest *k*_cat _value (289.1 S^-1^). Presumably the structure of ρ-nitrophenyl acetate is closer to the natural substrate of Sys410.

Sys410 efficiently degraded cyhalothrin, cypermethrin, sumicidin, deltamethrin under assay conditions of 37°C for 15 min, with exceeding 95% hydrolysis rate. Therefore, the pyrethroid-hydrolyzing esterase was capable of hydrolyzing a relatively wide range of compounds with similar chemical linkage at normal temperatures, which suggested that it possessed broader substrate specificities. This feature was similar to the pyrethroid hydrolases [15,16,18], while Sys410 displayed higher hydrolysis rate. However, this observation did not quite agree with data reported by Stok et al. [17].

## Conclusion

Due to the importance of discovering new biotechnology products to deal with pyrethroid residues, we identified a novel pyrethroid-hydrolyzing enzyme Sys410 belonging to familyV esterases/lipases with activity-based functional screening from Turban Basin metagenomic library. The soil has unique environmental characteristics with land surface temperature 82°C for a certain period. This is the first report to construct metagenomic libraries from Turban Basin to obtain the thermostable pyrethroid-hydrolyzing enzyme. The recombinant Sys410 with broad substrate specificities and high activity was the most thermostable one of the pyrethroid-hydrolyzing esterases studied before, which was necessary to fulfill the practical requirements of bioremediation to enable its use in situ for detoxification of pyrethroids. Further studies will supply important data, such as high density fermentation, for future application of the enzyme for promising environmental protection and food security.

## Materials and methods

### Chemicals and reagents

Cyhalothrin (98%), cypermethrin (98%), sumicidin (98%), deltamethrin (98%) were kindly provided by Zhong Shan Pesticide Factory (Guang dong, China). All ρ-nitrophenyl esters were purchased from Sigma. All other chemicals and reagents were of analytical grade and were purchased from commercial sources, unless otherwise stated.

### Bacterial strains and plasmids

*E. coli *TOP10 was used as the host for gene cloning and *E. coli *BL21 (DE3) (Novagen, Madison, USA) for protein expression. The pUC118 (TaKaRa, Dalian, China) and pET-28a (+) (Novagen, Madison, WI, USA) were used to construct metagenomic libraries and express the target protein, respectively. *E. coli *transformants were grown at 37°C in Luria-Bertani (LB) broth with appropriate antibiotics.

### DNA extraction from environmental samples

The topsoil samples (5 to 10 cm) from Turban Basin were used for the experiments. Samples were collected and stored at -80°C until the DNA extraction was performed. The total DNA was extracted based on a method described previously [38]. Routine DNA manipulations were carried out according to standard techniques. *Bam*HI, *Eco*RI, T4 DNA ligase and DNA polymerase were purchased from TaKaRa (Dalian, China). Each enzyme was used according to the recommendations of the manufacturer. Plasmids were prepared from *E. coli *by using E.Z.N.A Plasmid Mini Kit. DNA fragments were isolated from agarose gels by using E.Z.N.A Gel Extraction Kit.

### Screening for pyrethroid-hydrolyzing esterases from a metagenomic library and gene anlysis

The purified DNA was partially digested with *Bam*HI. DNA fragments of 2.5-10 kb were ligated into *Bam*HI-digested pUC118, and the ligated products were transformed into *E. coli *TOP10. The transformed cells were plated onto LB agar plates containing 50 μg/mL ampicillin, 0.5 mM isopropyl-β-D-thiogalactopyranoside (IPTG) and 100 μM 5-bromo-4-chloro-3-indolyl caprylate (X-caprylate). After incubation at 37°C for 24 h, clones with blue color were selected. Then clones with blue color were further tested for the ability to hydrolyze hydrolyze pyrethroids confirmed by gas chromatography analysis. Only one transformant with pyrethroid-hydrolyzing activity was obtained. Then the recombinant plasmid (pUC3) was sequenced on ABI 377 DNA sequencer. Sequence manipulation, open reading frame (ORF) searches, and multiple alignments among similar enzymes were conducted with Clustal W software. Database homology search was performed with BLAST program provided by NCBI. The conserved patterns of discrete amino acid sequences related enzymes were analyzed by Clustal W program.

### Cloning, expression and purification of pyrethroid-hydrolyzing esterase

The putative esterase gene was amplified by PCR with the pUC118-*sys410 *as template using primers which contained restriction enzyme sites *Bam*HI and *Eco*RI. Amplified DNA was digested by *Bam*HI/*Eco*RI, ligated into pET-28a (+) which was linearized by *Bam*HI/*Eco*RI, then transformed into *E. coli *BL21 (DE3) cells. *E. coli *cells transformed with this plasmid were plated onto LB agar containing 50 μg/mL kanamycin. Transformed cells were grown in a 250 mL flask containing 50 mL of LB (50 μg/mL kanamycin) at 37°C until the cell concentration reached OD_600 _of 1.0, then induced with 0.6 mM IPTG. After incubation at 37°C for 8 h with shaking at 220 rpm, cells were harvested by centrifugation (6000 g, 10 min) at 4°C and suspended in binding buffer (0.5 M NaCl, 5 mM imidazole, 20 mM Tris-HCl, pH 7.9). The cells were disrupted by sonication, and the supernatant was collected by centrifugation (13000 g, 10 min) at 4°C. The sample was loaded onto a Ni-NTA His·Bind column pre-equilibrated with binding buffer. Then the column was washed with binding buffer and washing buffer (0.5 M NaCl, 60 mM imidazole, 20 mM Tris-HCl, pH 7.9). Finally, the bound protein was eluted with eluting buffer (1 M imidazole, 0.5 M NaCl, 20 mM Tris-HCl, pH 7.9). The fractions containing the recombinant protein Sys410 were collected and stored at -20°C.

### Determination of molecular mass

The molecular mass of the denatured protein was determined by sodium dodecyl sulfate-polyacrylamide gel electrophoresis (SDS-PAGE). 12% SDS-PAGE was prepared by the method of Laemmli [39]. Proteins were stained with Coomassie brilliant blue G-250. The molecular mass of the enzyme subunit was estimated using protein marker as standards, Rabbit muscle phosphorylase B (97,200 Da), bovine serum albumin (66,409 Da), ovalbumin (44,287 Da), carbonic anhydrase (29,000 Da), Soybean Trypsin Inhibitor (20,100 Da), Hen egg white Lysozyme (14,300 Da).

### Effect of pH and temperature on activity and stability of recombinant Sys410

The optimum pH of recombinant Sys410 was measured using ρ-nitrophenyl acetate as a substrate at 40°C. The buffers (the final concentration of 50 mM) used for the measurement were as below: citric acid-NaOH buffer (pH 3.5-5.5); potassium phosphate buffer (pH 5.0-7.0); Tris-HCl buffer (pH 6.5-9.0), glycine-NaOH buffer (pH 8.5-10.0). Overlapping pH values were used to verify that there were no buffer effects on substrate hydrolysis. The pH stability was tested after incubation of the purified enzyme for 24 h at 30°C in the above different buffers. The optimum temperature was determined analogously by measuring esterase activity at pH 6.5 in the temperature range of 20-80°C. Temperature stability was measured by preincubation of the purified enzyme for different time in 50 mM potassium phosphate buffer (pH 6.5) at different temperatures. The storage stability of the recombinant enzyme Sys410 was determined by preincubation of the purified enzyme for 10 weeks in 50 mM potassium phosphate buffer (pH 6.5) at 4°C and 25°C.

### Analysis of Sys410 activity

The esterase activity against ρ-nitrophenyl acetate (ρNPE) was determined by measuring the amount of ρ-nitrophenol released by esterase-catalyzed hydrolysis. The substrate (ρNPE) (3 mg) with final concentration of 0.3 mg/mL was dissolved in 1 mL of isopropanol and mixed with 9 mL of 50 mM potassium phosphate buffer (pH 6.5) containing gum arabic (0.1%, w/v) and Triton X-100 (0.6%, w/v). The hydrolysis of substrate was performed at 55°C for 5 min in 50 mM potassium phosphate buffer (pH 6.5). The production of ρ-nitrophenol was monitored spectrophotometrically at 405 nm by Labsystems Dragon Wellscan MK3. One unit of enzyme activity was defined as the amount of enzyme that produced 1 μmol of ρ-nitrophenol per minute under these conditions. In each measurement, the effect of nonenzymatic hydrolysis of substrates was taken into consideration and subtracted from the value measured when the enzyme was added.

### Determination of substrate specificity and kinetic parameters

Substrate specificity against different ρ-nitrophenyl esters was determined using ρ-nitrophenyl acetate (C2), ρ-nitrophenyl butyrate (C4), ρ-nitrophenyl caprylate (C6), ρ-nitrophenyl caproate (C8), ρ-nitrophenyl decanoate (C10), ρ-nitrophenyl laurate (C12) as substrates, the activity was then measured described above at the final substrate concentration of 0.3 mg/mL separately.

The purified Sys410 was incubated with various concentrations of ρ-nitrophenyl acetate. The final concentration ranged from 1.0 mM to 10.0 mM in potassium phosphate buffer (6.5). The kinetic constants were calculated by fitting the initial rate data into the Michaelis-Menten equation using the GraFit software (version 6; Erithacus Software Ltd., Horley, UK).

### Gas chromatography analysis of different pyrethroids degradation by Sys410

Sys410 was tested for hydrolysis of cyhalothrin, cypermethrin, sumicidin, deltamethrin by Gas chromatography. The enzyme samples (24 μg) with 5 mg/mL substrates in 50 mM potassium phosphate buffer (pH 6.5) were incubated at 37°C for 15 min. Aliquots (1 μL) of the reaction mixtures were loaded onto a Gas chromatography system (GC-2010, Shimadzu Corporation, Japan) with ECD Detector using RestekRTX-5 column (30 m × 0.25 mm × 0.25 μm). The column flow was 2 mL/min. The column was set at 150°C for 1 min, and heated to 270°C at 30°C/min^-1^. The temperature of ECD Detector was 300°C. The electric current was 1 nA. In each measurement, the effect of nonenzymatic hydrolysis of substrates was taken into consideration and subtracted from the value measured when the enzyme was added.

### Nucleotide sequence accession number

The nucleotide sequence data reported here have been submitted to the nucleotide sequence databases under accession number (JQ272178).

## Competing interests

The authors declare that they have no competing interests.

## Authors' contributions

XJF: have performed construction of metagenomic library, gene cloning and expression in *E. coli*, and enzyme characterization. XLL: have collected samples in Turban Basin. RH: have extracted DNA from soil samples. YHL: have conceived the study, designed and supervised the experiments, drafted and revised the manuscript. All authors have read and approved the manuscript.
